# Association between social support and place of delivery: a cross-sectional study in Kericho, Western Kenya

**DOI:** 10.1186/1471-2393-13-214

**Published:** 2013-11-21

**Authors:** Mayo Ono, Akiko Matsuyama, Mohamed Karama, Sumihisa Honda

**Affiliations:** 1 Graduate School of Biomedical Sciences, Nagasaki University, 1-12-4 Sakamoto, Nagasaki 852-8523, Japan; 2 Graduate School of International Health Development, Nagasaki University, Nagasaki, Japan; 3 Centre for Public Health Research (CPHR), Kenya Medical Research Institute (KEMRI), Nairobi, Kenya

**Keywords:** Kenya, Maternal health, Place of delivery, Social support

## Abstract

**Background:**

An estimated 358,000 maternal deaths still occur worldwide each year. The place of delivery is of great significance to the reduction of maternal mortality. Moreover, socio-economic factors, cultural traits, and local customs are associated with health-seeking behavior. This study aimed to explore determinants of association between social support and place of delivery.

**Methods:**

This cross-sectional study was conducted from September to November 2011 at Sosiot Health Center, Kericho West District, Kenya. Participants were 303 mothers who brought their babies to the health center for immunization within their first year of life. Women underwent a structured interview using a questionnaire on demographic characteristics and their experiences of delivery including place of delivery and social support.

**Results:**

The proportion of deliveries at health facilities was significantly higher in unmarried than married women (93% and 78%, respectively; P = 0.008). Unmarried women whose mothers supported them in housework and whose sisters helped them fetch water were more likely to deliver at health facilities (P = 0.002 and 0.042, respectively) than those without this support. However, married women whose husbands supported them in farming and whose neighbors helped them fetch water were less likely to deliver at health facilities (P = 0.003 and 0.021, respectively) than those without this support. Married women who were advised to deliver at a health facility by their mother-in-law or health staff were more likely to deliver at health facilities (P = 0.015 and 0.022, respectively) than those who did not receive this advice. Multivariate analysis revealed that married women were more likely to deliver at health facilities if they were highly educated (odds ratio [OR] = 2.5); had financial capability (OR = 4.3); had medical insurance (OR = 4.2); were primiparous (OR = 3.5); did not have the support of sisters-in-law for fetching water (OR = 2.2); or were advised to deliver at a health facility by family or neighbors (OR = 2.5).

**Conclusions:**

Promotion of delivery at health facilities requires approaches that consider women’s social situation, since factors influencing place of delivery differ for married and unmarried women.

## Background

An estimated 358,000 maternal deaths still occur worldwide each year [1]. Most of these deaths occur during labor, delivery, or the first 24 hours postpartum, and most complications cannot be predicted or prevented [2]. Where women deliver, who attends them, and how quickly they can be transported to referral-level care are thus crucial factors in determining the ability to successfully intervene [3]. The general pattern of utilization of maternal health services contributes to the incidence of maternal mortality and morbidity [4]. However, many women do not have access to the services they need for various reasons. Moreover, socio-economic factors, cultural traits, and local customs are associated with health-seeking behavior. For instance, there are places where services are available but women do not make use of them [3]. Women may prefer to stay at home for a delivery so that they can take care of family members such as young children or elderly relatives and manage their daily household chores [5]. Previous studies have shown that several factors are predictors of women accessing health services [6,7]. It is important to identify which factors lead women to deliver at health facilities. Social support is also an important variable influencing health [8]. However, little attention has been paid to the possible association between social support and place of delivery. The aim of the present study was to determine the association between social support and place of delivery, and other factors influencing delivery location.

### Place of delivery and types of birth attendant in Kenya

Delivery with skilled birth attendants is critical to the reduction of maternal mortality [3]. In Kenya, 43.8% of laboring women were assisted by skilled birth attendants, and 42.6% delivered at health facilities between 2008 and 2009 [9]. The place of delivery, for example, health facility or home, is of great significance: home births are associated with increased maternal mortality, since women delivering at home do not have access to the professional assistance provided in facilities. In Kenya, the National Reproductive Health Policy (2007) has brought about a paradigm shift towards a focus on skilled birth attendants for all pregnant women, thus necessitating a policy change regarding traditional birth attendants (TBAs) as providers of delivery services [10]. However, TBAs continue to play a vital role in delivery, assisting with 27.6% of births. Moreover, relatives and friends assist with 21.2% of deliveries between 2008 and 2009 [9]. In Kericho, the location of the present study, the proportion of women delivering at a health facility remained at 50% in 2007 [11], a proportion considered inadequate.

### Social support

Social support from a spouse or partner and a social network of family and friends has the potential to influence women’s decisions regarding obtaining prenatal care [12]. As in most societies, women have traditionally relied on other women for social support during pregnancy, childbirth, and breastfeeding [13,14]. Female relatives and friends accompany laboring women to maternity units [15], and the presence of a female relative during labor is associated with improved labor outcomes [16].

Social support has been measured in numerous ways. One frequent criticism of research in this area is the lack of consensus about social support in terms of its definition and how best to measure it [17-19]. House, a sociologist, specified that potential forms of social support were emotional support, appraisal support, informational support, and instrumental support [18]. Schaefer classified social support into tangible, emotional, and informational support, and he focused on relationships between these types of social support [19]. A previous study found that social support is related to health behaviors [12], and absence of social support was associated with increased maternal mortality [20]. To obtain a better understanding of the effect of social support on place of delivery, the source and types of social support should be considered.

### The Kenyan context

Maternal mortality in Kenya remains high at 488 per 100,000 live births in 2008–09 [9]. Kericho District (comprising East and West Kericho) is located about 260 km from the capital, Nairobi. Kericho District has a population of 503,468 [21]. This district had nine hospitals, 11 health centers, five health clinics, one maternity nursing home, and 97 dispensaries [11]. The doctor/patient ratio was 1:15,000, and 40% of houses were located within 15 km from health facilities [21].

## Methods

### Subjects

This cross-sectional study was conducted from September to November 2011 at Sosiot Health Center, Kericho West, Kenya. Information was obtained by means of a structured interview using a questionnaire. Kericho was one of the target districts of two recent community health projects aimed at improving maternal and neonatal health. Those projects were implemented by the Kenyan Government in collaboration with the Japan International Cooperation Agency (JICA) and a Japanese non-profit organization, Health and Development Service (HANDS). Promotion of delivery at health facilities was included in the message of the one of those projects [11]. Sosiot Health Center was involved in both of these projects. The center is situated beside a major thoroughfare, and is a 15 to 20 minute car journey from the center of commerce in Kericho. The target population of this health center was 43,493 people [11], making it a larger scale center than other health facilities in this region.

### Questionnaire

The respondents were 306 mothers aged 18 to 49, who brought their babies to Sosiot Health Center for immunization within their first year of life, from September to November 2011. We excluded from the analysis two respondents who delivered on the way to the health facility and one respondent who did not answer the question on marital status. Thus data from 303 respondents (99%) were analyzed. Local people who were at least high school graduates and spoke English, Kiswahili (the national language), and Kipsigis (the dominant language spoken at the study site) fluently were hired as research assistants. They were trained by the researcher so that they conducted interviews smoothly and protected respondents’ privacy. The interviews were conducted at Sosiot Health Center. The questionnaire was constructed to ascertain the demographic characteristics of the subjects, as well as their experiences of delivery including place of delivery and social support (see Additional files 1, 2 and 3).

### Dependent variable

The primary dependent variable was the place of delivery of the latest child, dichotomized as health facility or home. Health facility deliveries included those at the dispensary, health center, sub-district hospital, district hospital, national hospital, and private hospital/clinic.

### Demographic and birth experience variables

We collected data relating to maternal age, infant age, tribe, education level, occupation, marital status, economic status, medical insurance, the time required to reach the nearest delivery facility, types of birth attendant for home delivery, and parity. Information on household assets (clock or watch, electricity, radio, television, mobile telephone, non-mobile telephone, refrigerator, and solar panel) was used to derive a wealth index using the Kenya DHS [9]. Respondents were categorized into three levels by the wealth index. The time required to travel to the nearest delivery facility was calculated in terms of total minutes by foot, bike, shared taxi, and private car or taxi. Respondents were then divided into two groups by the median travel time.

### Social support variables

Social support was measured in terms of: 1) Support for daily tasks (housework, fetching water, and farming), and 2) Advice to deliver in a health facility. Support for daily tasks indicated instrumental support, and advice regarding facility delivery reflected informational support. 1) Respondents were asked if they had support for housework, fetching water, and farming, and by whom. They chose from the following answers: husband, mother-in-law, mother, father-in-law, father, sisters-in-law, sisters, brothers-in-law, brothers, female relatives, children, friends, neighbors, domestic servants, co-wives, and others. 2) Respondents were asked whether somebody had advised them to deliver at a health facility. They chose from the following answers: husband, mother-in-law, mother, father-in-law, father, sisters-in-law, sisters, female relatives, children, friends, neighbors, domestic servants, co-wives, health staff, and others.

### Ethical considerations

All women who participated provided written informed consent after reading through the consent form with the interviewer. They were informed that they had the right to refuse participation or to withdraw from this study at any time without prejudice to themselves. This study was approved by the ethics committees of Nagasaki University (Nagasaki, Japan) in December 2010 and Kenya Medical Research Institute (Nairobi, Kenya) in July 2011.

### Data analysis

The chi-square test was used for nominal scale data, whereas the Cochran-Armitage trend test was used for ordinal scale data. The simultaneous effects of factors on facility delivery were analyzed using linear logistic models. The following factors were included in stepwise logistic regression: age, education level, occupation, economic status, medical insurance, the time required to travel to the nearest delivery facility, support of mother-in-law for household work, support of husband for farming, support of sisters-in-law for fetching water, advice on facility delivery from family or neighbors, advice on facility delivery from health staff, and parity. Odds ratios (OR) with 95% confidence intervals (CI) were calculated. IBM SPSS Statistics Version 21 was used for statistical analysis (SPSS Inc, Chicago, IL, USA).

## Results

Socio-demographic characteristics and birth experience are shown in Table 1. Among the 303 respondents, age ranged from 18 to 41 years and median age was 23 years. Around 86% of respondents had some primary education. The most common occupation was farming. About 97% of women were Kipsigis, and more than 80% of respondents were married. In their most recent delivery, 58 (19.1%) women delivered at home and 245 (80.9%) delivered at health facilities. Just less than half of women who delivered at home were assisted by their mother-in-law, and the next largest proportion was assisted by neighbors.

**Table 1:** Socio-demographic characteristics and birth experience (N = 303)

**Variable**		**Median**	**Range**
Maternal age (years)		23	18-41
Infant age (months)		4	0-12
		**N**	**%**
Tribe	Kipsigis	294	97.0
	Others	9	3.0
Education level	Lower primary	43	14.2
	Primary	124	40.9
	Vocational	1	0.3
	Secondary	105	34.7
	College	25	8.3
	University	5	1.7
Occupation	Farmer	251	82.8
	Self-employed	19	6.3
	Private employee	16	5.3
	Government employee	12	4.0
	Student	5	1.7
Marital status	Married/Cohabiting	245	80.9
	Unmarried	58	19.1
Economic status	0	6	2.0
(Household assets)	1	31	10.2
	2	64	21.1
	3	70	23.1
	4	52	17.2
	5	78	25.7
	6	2	0.7
Medical insurance	Yes	83	27.4
	No	219	72.3
	Unknown	1	0.3
Time to reach the nearest delivery facility	Less than 20 minutes	140	46.2
	20 minutes or more	163	53.8
Place of delivery	Health facility	245	80.9
	Home	58	19.1
Type of facility where delivery took place	Health center	147	60.0
	District hospital	69	28.2
	Private hospital/Clinic	24	9.8
	Dispensary	1	0.4
	Sub-district hospital	1	0.4
	National hospital	1	0.4
	Others	2	0.8
Type of birth attendant at home birth	Mother-in-law	26	44.8
	Neighbor	15	25.9
	Traditional birth attendant	4	6.9
	Sister-in-law	4	6.9
	Mother	2	3.4
	Grandmother	1	1.7
	Female relative	1	1.7
	Unknown	5	8.6
Parity	Primiparous	143	47.2
	Multiparous	160	52.8

Table 2 presents the associations between socio-demographic characteristics and place of delivery. Bivariate analysis indicated that facility delivery was more likely in younger women (P = 0.002), unmarried women (P = 0.008), primiparae (P < 0.001) and those with financial capability or high educational attainment (P < 0.001).

**Table 2:** Associations between socio-demographic characteristics and place of delivery (N = 303)

	**Home delivery**		**Facility delivery**		
	**(N = 58)**		**(N = 245)**		
	**N**	**%**	**N**	**%**	**P-value**
**Maternal age**					
18-25 (years)	31	14.4	184	85.6	
26-30 (years)	20	30.3	46	69.7	
≥31(years)	7	31.8	15	68.2	0.002^a^
**Education level**					
Primary or lower	47	28.1	120	71.9	
Secondary/Vocational	10	9.4	96	90.6	
College/University	1	3.3	29	96.7	<0.001^a^
**Occupation**					
Farmers	55	21.9	196	78.1	
Others	3	5.8	49	94.2	0.007^b^
**Marital status**					
Married/Cohabiting	54	22.0	191	78.0	
Unmarried	4	6.9	54	93.1	0.008^b^
**Economic status** (Household asset score)					
0-1	16	43.2	21	56.8	
2-3	31	23.1	103	76.9	
≥4	11	8.3	121	91.7	<0.001^a^
**Medical insurance**					
Yes	8	9.6	75	90.4	
No	50	22.8	169	77.2	0.009^b^
Unknown	0		1		
**Time to reach the nearest delivery facility**					
Less than 20 minutes	22	15.7	118	84.3	
20 minutes or more	36	22.1	127	77.9	0.16^b^
**Parity**					
Primiparous	13	8.1	147	91.9	
Multiparous	45	31.5	98	68.5	<0.001^b^

^a^Cochran-Armitage trend test ^b^Chi-square Test.

Table 3 shows associations between support for daily tasks and place of delivery among married women. Married women whose husbands supported them in farming and whose neighbors helped them fetching water were less likely to deliver at health facilities (P = 0.003 and P = 0.021, respectively) than those whose husbands and neighbors did not provide this support.

**Table 3:** Associations between support for daily tasks and place of delivery among married women

	**Support with housework**					**Support with fetching water**					**Support with farming**				
	**(N = 245)**					**(N = 231)**^**a**^					**(N = 229)**^**b**^				
	**Home delivery**		**Facility delivery**			**Home delivery**		**Facility delivery**			**Home delivery**		**Facility delivery**		
	**(N = 54)**		**(N = 191)**			**(N = 51)**		**(N = 180)**			**(N = 53)**		**(N = 176)**		
	**N**	**%**	**N**	**%**	**P-value**^**c**^	**N**	**%**	**N**	**%**	**P-value**^**c**^	**N**	**%**	**N**	**%**	**P-value**^**c**^
Support from mother-in-law															
Yes	29	20.4	113	79.6		17	20.0	68	80.0		5	20.0	20	80.0	
No	25	24.3	78	75.7	0.473	34	23.3	112	76.7	0.561	48	23.5	156	76.5	0.693
Support from husband															
Yes	3	27.3	8	72.7		1	5.0	19	95.0		36	31.6	78	68.4	
No	51	21.8	183	78.2	0.668	50	23.7	161	76.3	0.054	17	14.8	98	85.2	0.003
Support from sister-in-law															
Yes	17	25.0	51	75.0		21	29.6	50	70.4		4	40.0	6	60.0	
No^d^	37	20.9	140	79.1	0.489	30	18.8	130	81.3	0.067	49	22.4	170	77.6	0.196
Support from mother															
Yes	0	0.0	3	100.0		1	14.3	6	85.7		0	0.0	1	100.0	
No	54	22.3	188	77.7	0.354	50	22.3	174	77.7	0.614	53	23.2	175	76.8	0.582
Support from sister															
Yes	5	19.2	21	80.8		3	11.5	23	88.5		0	0.0	5	100.0	
No^e^	49	22.4	170	77.6	0.715	48	23.4	157	76.6	0.169	53	23.7	171	76.3	0.215
Support from neighbor															
Yes	8	36.4	14	63.6		26	30.2	60	69.8		12	27.3	32	72.7	
No	46	20.6	177	79.4	0.089	25	17.2	120	82.8	0.021	41	22.2	144	77.8	0.47

^a^Excluded women who did not have to fetch water because they had tap water.

^b^Excluded women who did not have to do farming because they had no farm or livestock.

^c^Chi-square test.

^d^Included women who did not have any sisters-in-law and who therefore did not have support from sisters-in-law.

^e^Included women who did not have any sisters and who therefore did not have support from sisters.

Table 4 shows associations between support for daily tasks and place of delivery among unmarried women. Unmarried women whose mothers supported them in housework and those whose sisters helped them fetch water were significantly more likely to deliver at health facilities (P = 0.002, P = 0.042, respectively) than those whose mothers and sisters did not provide this support.

**Table 4:** Associations between support for daily tasks and place of delivery among unmarried women

	**Support with housework (N = 58)**					**Support with fetching water (N = 58)**^**a**^					**Support with farming (N = 58)**^**b**^				
	**Home delivery**		**Facility delivery**			**Home delivery**		**Facility delivery**			**Home delivery**		**Facility delivery**		
	**(N = 4)**		**(N = 54)**			**(N = 4)**		**(N = 54)**			**(N = 4)**		**(N = 54)**		
	**N**	**%**	**N**	**%**	**P-value**^**c**^	**N**	**%**	**N**	**%**	**P-value**^**c**^	**N**	**%**	**N**	**%**	**P-value**^**c**^
Support from mother															
Yes	1	2.1	47	97.9		2	5.3	36	94.7		2	6.7	28	93.3	
No	3	30.0	7	70.0	0.002	2	10.5	17	89.5	0.463	2	8.0	23	92.0	0.85
Unknown	0		0			0		1			0		3		
Support from sister															
Yes	1	6.7	14	93.3		0	0.0	28	100.0		0	0.0	14	100.0	
No^d^	3	7.0	40	93.0	0.967	4	13.8	25	86.2	0.042	4	9.8	37	90.2	0.225
Unknown	0		0			0		1			0		3		
Support from neighbor															
Yes	0	0.0	3	100.0		2	13.3	13	86.7		0	0.0	11	100.0	
No	4	7.3	51	92.7	0.628	2	4.8	40	95.2	0.265	4	9.1	40	90.9	0.299
Unknown	0		0			0		1			0		3		

^a^Excluded women who did not have to fetch water because they had tap water.

^b^Excluded women who did not have to do farming because they had no farms or livestock.

^c^Chi-square test.

^d^Included women who did not have any sisters and who therefore did not have support from sisters.

Table 5 shows the associations between advice regarding facility delivery and place of delivery among married women. Married women who were advised by their mothers-in-law or by health staff to deliver at a health facility were more likely to do so (P = 0.015, P = 0.022, respectively) than those who were not.

**Table 5:** Associations between advice on facility delivery and place of delivery among married women

	**Home delivery**		**Facility delivery**		
	**(N = 54)**		**(N = 191)**		
	**N**	**%**	**N**	**%**	**P-value**^**a**^
Advised by mother-in-law					
Yes	5	9.6	47	90.4	
No	49	25.4	144	74.6	0.015
Advised by father-in-law					
Yes	1	100.0	0	0.0	
No	53	21.7	191	78.3	0.059
Advised by husband					
Yes	7	14.9	40	85.1	
No	47	23.7	151	76.3	0.189
Advised by sister-in-law					
Yes	0	0.0	3	100.0	
No^b^	54	22.3	188	77.7	0.354
Advised by mother					
Yes	1	10.0	9	90.0	
No	53	22.6	182	77.4	0.348
Advised by father					
Yes	0		0		
No	54		191		
Advised by sister					
Yes	1	33.3	2	66.7	
No^c^	53	21.9	189	78.1	0.653
Advised by neighbor					
Yes	2	40.0	3	60.0	
No	52	21.7	188	78.3	0.328
Advised by health staff					
Yes	27	17.4	128	82.6	
No	27	30.0	63	70.0	0.022

^a^Chi-square test.

^b^Included women who did not have any sisters-in-law and who were therefore not advised to have a facility delivery by their sisters-in-law.

^c^Included women who did not have any sisters and who were therefore not advised to have a facility delivery by their sisters.

Table 6 shows the associations between advice regarding facility delivery and place of delivery among unmarried women. There were no statistically significant differences between those who were advised to deliver at a health facility and those who were not.

**Table 6:** Associations between advice on facility delivery and place of delivery among unmarried women

	**Home delivery**		**Facility delivery**		
	**(N = 4)**		**(N = 54)**		
	**N**	**%**	**N**	**%**	**P-value**^**a**^
Advised by mother					
Yes	1	4.3	22	95.7	
No	3	8.6	32	91.4	0.535
Advised by father					
Yes	0	0.0	1	100.0	
No	4	7.0	53	93.0	0.784
Advised by sister					
Yes	0	0.0	1	100.0	
No^b^	4	7.0	53	93.0	0.784
Advised by neighbor					
Yes	0	0.0	1	100.0	
No	4	7.0	53	93.0	0.784
Advised by health staff					
Yes	2	7.1	26	92.9	
No	2	6.7	28	93.3	0.943

^a^Chi-square test.

^b^Included women who did not have any sisters and who were therefore not advised to have a facility delivery by their sisters.

Table 7 summarizes the results of multivariate logistic regression analysis of factors associated with the place of delivery in married women. Facility delivery was more likely among married women who were highly educated (OR = 2.5, CI: 1.0 − 6.1); who had financial capability (OR = 4.3, CI: 1.6 − 11.3); who had medical insurance (OR = 4.2, CI: 1.4 − 12.4); or who did not have previous birth experience (OR = 3.5, CI: 1.5 − 8.5). Facility delivery was also more likely among married women who did not have the support of sisters-in-law for fetching water (OR = 2.2, CI: 1.0 − 4.7), or who were advised to deliver at a health facility by family or neighbors (OR = 2.5, CI: 1.2 − 5.5).

**Table 7:** Logistic regression analysis of factors associated with place of delivery among married women (N =245)

**Variable**	**OR**^**a**^	**95% CI**^**b**^
Education level		
0: Primary and lower		
1: Secondary/Vocational and above	2.5	1.0- 6.1
Economic status (Household asset score)		
0: ≤3		
1: ≥4	4.3	1.6-11.3
Medical insurance coverage		
0: No		
1: Yes	4.2	1.4-12.4
Parity		
0: Multiparous		
1: Primiparous	3.5	1.5- 8.5
Sister-in-law’s support for fetching water		
0: Yes		
1: No	2.2	1.0- 4.7
Advice from family or neighbor to deliver at a health facility		
0: No		
1: Yes	2.5	1.2- 5.5

All variables were included in the logistic regression model.

^a^OR = odds ratio.

^b^CI = confidence interval.

## Discussion

The present study found that more than 80% of women delivered at health facilities, and less than 20% delivered at home. This is a high proportion of facility deliveries and low proportion of home deliveries in comparison to findings of the Kenya DHS (national estimate, 42.6% facility deliveries and 56.2% home deliveries) [9]. We believe this is because a large public health intervention focusing on maternal health had recently involved the current study site, thereby increasing the proportion of facility deliveries. Moreover, respondents were mothers who brought their babies to the health center for immunization. Thus we might have selected those who had already easy access to health facilities. However, a high proportion of facility deliveries is not unusual in urban areas within sub-Saharan Africa. Progress towards the professionalization of childbirth attendance in the urban population was already good in 1992 (>70% of births were attended by health professionals) and even better in 2000 [3]. A similar rise in facility deliveries would have accompanied this, since most professional assistance is provided in health facilities. On the other hand, rural sub-Saharan Africa showed no improvement; 32% of deliveries were attended by health professionals in the early 1990s and this proportion remained the same in 2000 [3]. While the present study site is not located in an area as urbanized as Nairobi, the capital of Kenya, some of its characteristics are more similar to urban areas than to rural areas because of economic development accompanying the tea industry.

Bivariate analysis showed that place of delivery was associated with age, educational level, occupation, marital status, economic status, medical insurance cover, parity, support for daily tasks, and advice regarding facility delivery. Many studies have investigated factors influencing place of delivery. For instance, a study in Kenya [22] and one in Burkina Faso [23] reported that a high education level was associated with a high proportion of facility deliveries. Another study in African countries including Kenya showed that favorable economic status was a significant predictor of the proportion of facility deliveries [7]. Moreover, the present study found that women who had medical insurance were more likely to deliver at health facilities, in agreement with a previous study conducted in Kenya [24]. In addition, primiparae were significantly more likely than multiparae to deliver at health facilities, as in previous studies [25]. Women who have not previously given birth tend to be more worried about complications than those who have had previous deliveries; therefore, they tend to choose facility delivery.

The current findings showed that types of support and the people providing this support were associated with place of delivery. Multivariate logistic regression analysis revealed that married women whose sisters-in-law helped them fetch water were less likely to deliver at health facilities. Bivariate analysis revealed that married women whose husbands supported them in farming and those whose neighbors helped them fetch water were also less likely to deliver at health facilities. These results are explained by the local cultural context. In the area of the study, women usually live near their husband’s family after marriage, and mutual aid and collaboration with neighbors is quite common [26]. Having support from sisters-in-law, a husband or neighbors indicates stable relationships within the marriage, and with the husband’s family and community. Our results showed that 44.8% of women who delivered at home were assisted by their mother-in-law (not TBA). Married women who had stable relationships with their in-laws readily received support to deliver at home, thereby decreasing their rate of facility delivery. Moreover, if a member of their husband’s family recommends a home delivery to a married woman, it is difficult to act against such advice, since married women are under pressure from this side of the family. This supports the previously reported idea that social support can include negative support as well as positive support [27]. Some married women deliver at health facilities because of positive reasons such as believing it is safer than home delivery. A previous study found that the perceived competency of midwives and better equipment were among the reasons women used childbirth services [5]. On the other hand, some married women who do not have a good relationship with their husbands or their neighbors might deliver at health facilities for negative reasons such as not having support for home delivery. Hence, facility delivery may be chosen for both positive and negative reasons.

The present study found that unmarried women whose mothers supported them in housework and whose sisters helped them fetch water were more likely to deliver at health facilities. Unmarried women are of course not subject to pressure from a husband’s family, and they can readily get support from their original family, so they are less likely to be influenced by traditional custom related to home delivery. As a result, unmarried women who had social support were more likely to deliver at health facilities.

The present multivariate analysis revealed that married women who were advised to deliver at a health facility by family or neighbors were more likely to do so than those who did not receive such advice. Bivariate analysis also showed that married women who were given such advice by their mother-in-law were more likely to deliver at a health facility than those who did not receive such advice. This suggests married women are more likely to be affected by advice from their mother-in-law than from any other family members. Mothers-in-law appear to be very influential regarding where their daughters-in-law deliver, and should therefore be involved with promotion of facility delivery. Moreover, married women who were advised by health staff to deliver at a health facility were more likely to do so than those who did not receive such advice, in agreement with a previous study [28]. However, the fact that women could receive advice from health staff suggests they already had good access to health services. Indeed, a previous study reported that professionals such as health care providers and counselors were not considered sources of social support by women [12]. Therefore, promotion of facility delivery should not accordingly be implemented only by health staff but also by close kinship.

### Limitations of the study

Several limitations are worth noting. First, the respondents were mothers who brought their babies to the health center, raising the possibility of selection bias. Second, participants might have had recall bias about pregnancy and childbirth. Furthermore, women could have responded with socially desirable answers such as reporting that they considered facility delivery in a positive light. In addition, ascertaining support for housework, fetching water, and farming, and advice regarding facility delivery does not provide the whole picture of social support. We therefore measured only some of the instrumental and informational support available to women. Finally, we conducted multivariate analysis only for married women, because the number of unmarried women was inadequate. The generalizability of our findings may accordingly be limited.

## Conclusions

In conclusion, our study found that social support was associated with place of delivery. This support took the form of instrumental support, such as support for daily tasks, and informational support, such as advice to deliver in a health facility. However, married and unmarried women differed in terms of the factors influencing their decision about where to deliver. Married women who had instrumental support were less likely to deliver at health facilities than those without this support. In contrast, unmarried women who had instrumental support were more likely to deliver at health facilities than those without this support. Married women who had informational support were also more likely to deliver at health facilities than those without such support. A woman’s mother-in-law was the most influential person regarding place of delivery. We therefore need to recruit as advocates not only women of reproductive age but also those in the same generation as their mothers-in-law. These findings can be used by policy makers, planners, and health care professionals to take into account social support issues in improving health facility deliveries. Increasing the awareness of women and their family members about the benefits of facility deliveries are recommended.

## Competing interests

The authors declare that they have no competing interests.

## Authors’ contributions

MO conceived of the study, did the data collection and analysis, wrote the paper. SH and AM also wrote the paper. SH and MK contributed to the study design. All authors read and approved the final manuscript.

## Pre-publication history

The pre-publication history for this paper can be accessed here:

http://www.biomedcentral.com/1471-2393/13/214/prepub

## Supplementary Material

Additional file 1 Questionnaire1: questions about socio-demographic characteristics. Click here for file

Additional file 2 Questionnaire2: questions about birth experience. Click here for file

Additional file 3 Questionnaire 3: questions about social support. Click here for file
