# Supplementary material for: Association between social support and place of delivery: a cross-sectional study in Kericho, Western Kenya
Source: BMC Pregnancy Childbirth. 2013 Nov 21;13:214. doi: 10.1186/1471-2393-13-214 (PMC4222494; doi:10.1186/1471-2393-13-214)
Supplement: Additional file 1 — Questionnaire1: questions about socio-demographic characteristics. [file 1471-2393-13-214-S1.pdf]

Questionnaire 1 (Socio-demographic characteristics)

Q1	Participant's Address	1	Kericho West (Belgut) District
		2	Kericho (East) District
		3	Others/specify ( )
Q2	Last baby's age	( ) months	
Q3	How old are you?	( ) years old	
Q4	Have you ever attended school?	Yes No	
Q5	If "Yes" in Q4, What is the highest level of education you attended: primary, vocational, secondary, or higher?	1	Less than Primary
		2	Primary
		3	Post-Primary/Vocational
		4	Secondary/'A' LEVEL
		5	College (Middle Level)
		6	University
		7	Others/Specify ( )
Q6	What is your main work?	1	Farmer for own consumption
		2	Farmer for commercial products
		3	Government employee
		4	Private employee
		5	Self-employed
		6	School student
		7	Others/specify ( )
Q7	What is your ethnic group/tribe?	1	Kipsigis
		2	Others/Specify ( )
Q8	Which of these assets are found in your household?		
Q8-1	Clock or watch	Yes No	
Q8-2	Electricity	Yes No	
Q8-3	Radio	Yes No	
Q8-4	Television	Yes No	
Q8-5	Mobile telephone	Yes No	
Q8-6	Non-mobile telephone	Yes No	
Q8-7	Refrigerator	Yes No	

Q8-8	Solar panel	Yes No	
Q9	Do you have any medical insurance?	1	NHIF (National Hospital Insurance Fund)
		2	Private Insurance
		3	No, I don't have.
Q10	What is your current marital status?	1	Married /Cohabiting
		2	Divorced
		3	Widowed
		4	Never married / Never lived together
		5	Others/Specify ( )
