# Supplementary material for: Association between social support and place of delivery: a cross-sectional study in Kericho, Western Kenya
Source: BMC Pregnancy Childbirth. 2013 Nov 21;13:214. doi: 10.1186/1471-2393-13-214 (PMC4222494; doi:10.1186/1471-2393-13-214)
Supplement: Additional file 2 — Questionnaire2: questions about birth experience. [file 1471-2393-13-214-S2.pdf]

Questionnaire 2 (Birth experience)

Q1	Where did you give birth to your last child?	HEALTH FACILITY	
		1	Health Center
		2	Dispensary
		3	Sub-District Hospital
		4	District Hospital
		5	Private Hospital/Clinic
		NOT HEALTH FACILITY	
		6	Home
		7	On the way to health facility
		8	Others/Specify ( )
Q2	If you chose 6 (gave birth at home) in Q1, answer the following question.<br>Who was the main person to assist you during your last child birth?<br>*The person who handled the baby after it came out.	1	Traditional birth attendant
		2	Mother-in-law
		3	Mother
		4	Sister-in-law
		5	Sister
		6	Female relative<br>Specify ( )
		7	My child
		8	Friend
		9	Neighbor
		10	Others/Specify ( )
Q3	Have you ever delivered before this baby?	Yes No	
Q4	How long does it take from your home to the nearest delivery facility?	1	( ) minutes by foot
		2	( ) minutes by bike
		3	( ) minutes by matatu
		4	( ) minutes by taxi or private car
		5	( ) minutes by Others/Specify ( )
	*Calculate total minutes.	Total ( ) minutes	
