# Supplementary material for: Association between social support and place of delivery: a cross-sectional study in Kericho, Western Kenya
Source: BMC Pregnancy Childbirth. 2013 Nov 21;13:214. doi: 10.1186/1471-2393-13-214 (PMC4222494; doi:10.1186/1471-2393-13-214)
Supplement: Additional file 3 — Questionnaire 3: questions about social support. [file 1471-2393-13-214-S3.pdf]

Questionnaire 3 (Social support)

Q1	When you were unwell during pregnancy or when you needed to go for medical care including Antenatal care (ANC), were there people who helped you in fetching water?	1	Husband
		2	Mother-in-law
		3	Mother
		4	Father-in-law
		5	Father
		6	Sister-in-law
		7	Sister
		8	Brother in law
		9	Brother
		10	Female relative
		11	Children
		12	Friends
		13	Neighbors
		14	House girl or house boy
		15	Co-wife
		16	Others/specify ( )
		17	Nobody
Q2	When you were unwell during pregnancy or when you needed to go for medical care including Antenatal care (ANC), were there people who helped you in household chore (excluding fetching water)?	1	Husband
		2	Mother-in-law
		3	Mother
		4	Father-in-law
		5	Father
		6	Sister-in-law
		7	Sister
		8	Brother in law
		9	Brother
		10	Female relative
		11	Children
		12	Friends
		13	Neighbors
		14	House girl or house boy
		15	Co-wife
		16	Others/specify ( )
		17	Nobody

Q3	When you were unwell during pregnancy or when you needed to go for medical care including Antenatal care (ANC), were there people who helped you in farming?	1	Husband
		2	Mother-in-law
		3	Mother
		4	Father-in-law
		5	Father
		6	Sister-in-law
		7	Sister
		8	Brother in law
		9	Brother
		10	Female relative
		11	Children
		12	Friends
		13	Neighbors
		14	House girl or house boy
		15	Co-wife
		16	Others/specify ( )
		17	Nobody
Q4	Did health staff advised you to deliver at health facility during (any of) your Antenatal care (ANC) visit(s) of your last pregnancy?	Yes	No
Q5	Did somebody apart from health staff advised you to deliver at health facility?	Yes	No
Q6	If "Yes" in Q5, who advised you?	1	Husband
		2	Mother-in-law
		3	Mother
		4	Father-in-law
		5	Father
		6	Sister-in-law
		7	Sister
		8	Female relative
		9	Children
		10	Friends

		11	Neighbors
		12	House girl or house boy
		13	Co-wife
		14	Others/specify ( )
